# Effects of repetitive transcranial magnetic stimulation combined with cognitive training on cognitive function in patients with Alzheimer’s disease: a systematic review and meta-analysis

**DOI:** 10.3389/fnagi.2023.1254523

**Published:** 2024-01-25

**Authors:** Gaotian Liu, Bing Xue, Yafei Guan, Xianwu Luo

**Affiliations:** ^1^Wuhan University School of Nursing, Wuhan, China; ^2^Department of International Medical Services, Peking Union Medical College Hospital, Chinese Academy of Medical Sciences, Beijing, China

**Keywords:** Alzheimer’s disease, cognitive function, repetitive transcranial magnetic stimulation, cognitive training, meta-analysis

## Abstract

**Purpose:**

To evaluate the effect of repetitive transcranial magnetic stimulation (rTMS) paired with cognitive training on cognitive function in Alzheimer’s Disease (AD) patients.

**Methods:**

PubMed, The Cochrane Library, Embase, CINAHL Complete (EBSCO), China National Knowledge Infrastructure (CNKI) and WanFang Database were searched. The risk of bias was appraised through the Cochrane collaboration tool. A meta-analysis was conducted, including an assessment of heterogeneity.

**Results:**

Ten studies comprising 408 participants were included. The addition of rTMS significantly improved overall cognition in patients compared with cognitive intervention alone (*p* < 0.05 for all tests). The treatment also had some continuity, with significant improvements in cognitive function within weeks after the treatment ended (*p* < 0.05 for all tests).

**Conclusion:**

Repetitive transcranial magnetic stimulation combined with cognitive training (rTMS-CT) is a valuable technique for the cognitive rehabilitation of AD patients. It is beneficial to improve the cognitive ability of patients and restore their overall functional state. The results of the study may provide a basis for clinical providers to implement interventions that facilitate the design of more rigorous and high-quality interventions.

**Limitations:**

The number of studies and sample size in our study were small. We did not explore possible interactions between rTMS and medications and mood improvement after rTMS due to inadequate data.

**Systematic review registration:**

This study was registered on PROSPERO with registration number CRD42023405615.

## 1 Introduction

Alzheimer’s disease (AD) is a prevalent neurological illness in older adults that is defined by a gradual deterioration in cognition, behavior, and everyday living ability, accounting for around 60–80% of dementia causes (Knopman, 2011). The pathophysiology of AD is complex and involves a variety of factors, including beta-amyloid plaques, neuro progenitor fibril tangles, and inflammation. These factors lead to neuronal loss, synaptic dysfunction, and disruption of neural networks, resulting in cognitive impairment and dementia (Scheltens et al., 2021). Cognitive decline is considered the earliest symptomatic manifestation of AD (Rabin et al., 2017). Cognitive decline is associated with lower volume in the medial temporal lobes (including the hippocampus) and other AD-related cortical regions (Sanchez-Benavides et al., 2018). The number of cases of Alzheimer’s disease has grown as the population has aged. By 2050, the number of Alzheimer’s sufferers is predicted to reach 152 million (Vecchio et al., 2022). Current studies suggest that AD is incurable, and in recent years, the onset age of AD tends to be younger (Jian et al., 2020). This has undoubtedly led to substantial medical expenses. AD has become a serious global issue, and it is imperative to understand its pathology and explore new therapies.

At present, the mainstream treatment for AD is a drug intervention. Drug therapy attempts to cure it by reducing β-amyloid deposition and neurofibrillary tangles while boosting cholinergic nerve function and excitatory neurotransmitters (Guo et al., 2015). However, due to the complex pathogenesis, drug intervention only treats its symptoms (Yin et al., 2022). The need for non-pharmacological interventions such as is becoming increasingly urgent as patients suffer physical pain and social burdens. It was reported that the dietary-and bioactive compounds-based approaches, exercise and some complementary/alternative medicine techniques were related to a healthy aging, mental health and cognitive status (Lanza et al., 2018; Fisicaro et al., 2021, 2022; Currenti et al., 2023; Godos et al., 2023). Even if these factors do not directly affect the pathology of Alzheimer’s disease, they can make a difference for people with Alzheimer’s disease (Scheltens et al., 2021).

Repetitive transcranial magnetic stimulation (rTMS) is a non-invasive stimulation of the brain with a favorable prognosis for patients with psychiatric and neurological disorders by regulating cortical excitability (Kobayashi and Pascual-Leone, 2003). In patients with cognitive decline, rTMS is often used to improve cognitive function, mood, and other symptoms. Studies have shown that rTMS can improve cognitive function by altering blood flow and neurotransmitter levels in the brain to affect the activity of neurons (Siebner and Rothwell, 2003). There is also a potential diagnostic and therapeutic role for rTMS in vascular dementia and other secondary dementias (Cantone et al., 2020; Lanza et al., 2022). It enables the assessment of motor domains, corticospinal tracts, and neurotransmission pathways in a variety of neurologic and neuropsychiatric disorders, including cognitive impairment and dementia (Di Lazzaro et al., 2021). Motor cortex excitability is a measure of how easily neurons in the motor cortex are activated to produce movement. It is usually assessed using TMS, which involves applying brief magnetic pulses to the scalp above the motor cortex so that an electric current flows through the brain tissue below and activates neurons. In patients with dementia, there is evidence of altered motor cortex excitability (Di Lazzaro et al., 2004). The stimulation settings of rTMS are equally important in terms of therapeutic effects (Gao et al., 2023). Repetitive TMS can improve cognition by activating particular cortical regions, such as the dorsolateral prefrontal cortex (DLPFC) (Alvarez-Salvado et al., 2014). Specifically, rTMS can increase neural excitability or inhibition in the target region, depending on the applied magnetic field strength and frequency. Low-frequency rTMS (≤1 Hz) decreases cortical excitability, whereas high-frequency rTMS (≤5 Hz) promotes it (Dong et al., 2018). In addition, rTMS may also improve cognitive function by promoting neuroplasticity. Neuroplasticity refers to the brain’s ability to adapt to environmental changes, including functions such as learning, memory, and recovery. The study found that rTMS can promote neuroplasticity by enhancing the connectivity and efficiency of neural networks (Lin et al., 2019).

Cognitive training (CT) is a safe, low-cost and widely used intervention. It is a common non-drug intervention for treating AD and aims to maintain cognitive ability in older adults. It is considered an important adjuvant or alternative therapy for drug intervention (Bahar-Fuchs et al., 2013; Hill et al., 2017). CT can focus on many domains of cognition, such as memory, attention and executive processing. It can be in the form of paper-and-pencil training or computerized training. By applying computerized training methods, the training difficulty can be selected according to the participants’ cognitive performance, and the training methods can be adjusted dynamically according to the training performance to achieve adaptive training effects. Currently, most cognitive domains are considered to be plastic. That is, training in a cognitive domain can improve performance in the same cognitive domain. In addition, research has found that the effects of cognitive training can be transferable, and training on one cognitive domain can improve both the performance of that cognitive domain and other cognitive domains (Qijuan et al., 2022; Vecchio et al., 2022). Repetitive transcranial magnetic stimulation combined with cognitive training (rTMS-CT), a novel intervention for AD, is practical (Leung et al., 2015; Gao et al., 2023).

Previous studies have found that the rTMS-CT to have a more positive impact on overall cognitive ability, executive function, working memory, and ability to perform daily activities (Sabbagh et al., 2020; Gao et al., 2023). As an emerging, safe and effective non-drug intervention for treating AD, rTMS-CT has been widely used, but the current research results are varied. Therefore, although rTMS-CT has shown a therapeutic prospect as a treatment for AD, it still needs further development. This study aimed to investigate the effects of rTMS-CT on cognitive function in patients with Alzheimer’s disease.

## 2 Materials and methods

A systematic review and meta-analysis was conducted according to the reporting checklist of the Preferred Reporting Items for Systematic Reviews and Meta-Analysis (PRISMA) (Liberati et al., 2009). This study was registered on PROSPERO on December 2023, with registration number CRD42023405615.

### 2.1 Search strategy

To include studies that met the criteria, we conducted a comprehensive search of the following databases from inception to January 2023: PubMed, the Cochrane Library, Embase and CINAHL Complete (EBSCO); and some Chinese databases, including China National Knowledge Infrastructure (CNKI) and WanFang. The search terms were “Alzheimer’s disease” OR “altimeters” OR “Alzheimer” OR “Alzheimer’s” OR “dementia” OR “related dementia” OR “AD” AND “repetitive transcranial magnetic stimulation” OR “rTMS” AND “cognitive training” OR” executive function training” OR “brain training” OR “CT.” References to recognized studies were manually searched as well. The searches were limited to human trials, with the entire text provided in English and Chinese.

### 2.2 Inclusion and exclusion criteria

Two investigators separately reviewed the eligibility of the literature; disagreements were addressed by consensus. Studies were considered if they met the following criteria: (1) participants were older adults with AD; (2) the intervention group received rTMS-CT; (3) randomized controlled trials; (4) the outcomes included the cognitive function. Animal studies, duplicate papers, studies including other therapies (such as tDCS), studies with insufficient data, and studies for which the corresponding author did not respond after being contacted were all disqualified.

### 2.3 Data extraction

Three reviewers were involved in data extraction. Two reviewers independently extracted data in a pre-designed form. The form included the following information: study characteristics (authors, year of publication, and journal name), participants (sample size, intervention type), and cognitive performance (ADAS-Cog or MMSE). Disagreements encountered during the screening process were resolved through mutual consultation or discussion with a third reviewer.

### 2.4 Risk of bias

Two reviewers independently used the Cochrane Collaboration to evaluate the quality of included studies (Higgins et al., 2011). The risk of bias was rated as low, high, or unclear. The assessment items include (a) Random sequence generation; (b) Allocation concealment; (c) Blinding and personnel, blinding of outcome assessment; (d) Approach for handling incomplete outcome data; (e) Selective reporting and other bias.

### 2.5 Data analysis

The Review Manager software version 5.4 was used to analyse all data from included studies. Effect sizes were calculated using the change in mean and standard deviation (SD) values (the difference between the latest follow-up and baseline scores). As effect estimates in pooled studies, mean differences (MD) or standardized mean differences (SMD) and their 95% confidence intervals (CI) were utilized. When the same result was examined with the same instrument, mean differences were used, while SMDs were used when the same outcome was tested with separate instruments.

Heterogeneity was assessed by the Cochrane Q statistic and the I^2^ statistic. Significant heterogeneity was considered if the value of *p* <0.10 or the I^2^ ≥ 50% (Higgins and Thompson, 2002). A fixed-effects model was used to pool the data for substantial heterogeneity; otherwise, a random-effects model was used. Subgroup analyses were conducted to explore possible heterogeneous resources for different comparator interventions. Sensitivity analyses were carried out by removing each study and recalculating the pooled estimates for the studies that remained. If the number of included studies was greater than 10, the funnel plot and Egger’s test were used to examine potential publication bias (Egger et al., 1997). The significance level was set at a value of *p* <0.05 (two-tailed).

## 3 Results

### 3.1 Study selection

**Figure 1:**
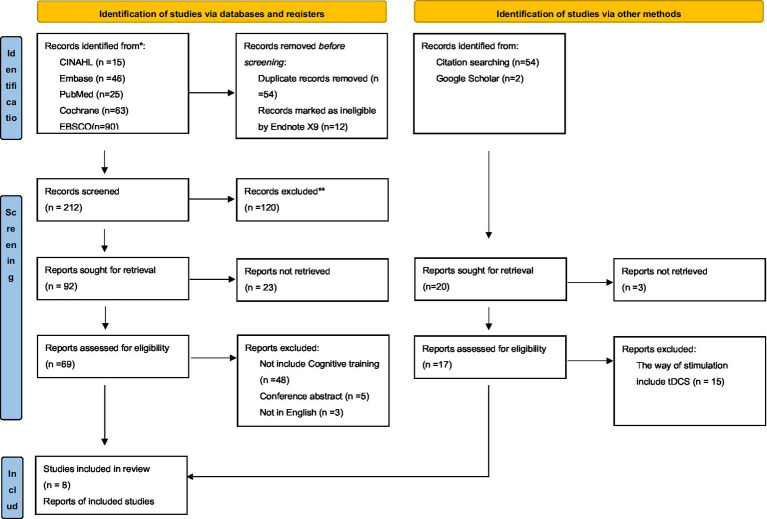
PRISMA 2020 flow diagram for new systematic reviews which included searches of databases, registration platform and other sources.

An electronic database search provided 278 results, and 212 records were retained after eliminating duplicate and unqualified study types using Endnote X9. From these records, 23 were eliminated by screening the titles and abstracts for potential inclusion; leaving 69 articles for secondary full-text examination. Further screening yielded 8 studies meeting the inclusion criteria. Two additional records were identified by a secondary search using Citation and Google Scholar. Ultimately, 10 articles (14 studies) were included in this study (Brem et al., 2013, 2020; Rabey et al., 2013; Lee et al., 2016; Zhang et al., 2019, 2022; Bagattini et al., 2020; Jiang et al., 2022; Qijuan et al., 2022; Vecchio et al., 2022). The flow chart shows the screening process (Figure 1).

### 3.2 Studies characteristics

The sample size varied from 12 to 80, and 221 participants who received rTMS and 187 who received sham stimulation were included in the meta-analysis. Ten of the fourteen studies used ADAS-Cog to assess cognitive function improvement, and nine used MMSE. The characteristics, stimulation with rTMS and cognitive training interventions of the fourteen selected studies are summarized in Table 1.

**Table 1:** Characteristics of included studies (*n* = 14).

Authors	Type of study	Subject	Intervention frequency/duration	Stimulation position/intensity	Coil
Brem et al. (2013)	RCT	Patients with MCI	7 sessions, weekly/6 weeks	DLPFC, L IFG, L STG/10 Hz	Figure-of-eight 70 mm air-cooled coil
Brem et al. (2020)	RCT	Patients with AD	5 sessions, weekly/6 weeks	DLPFC, L IFG, L STG, IPL/10 Hz	Figure-of-eight focal coil
Bagattini et al. (2020)	RCT	Patients with amnesic MCI or mild to moderate probable AD	5 sessions, weekly/4 weeks	lDLPFC/20 Hz, 90%	Figure-of-eight 70 mm air-cooled coil
Jiang et al. (2022)	RCT	Patients with AD	6 sessions, weekly/4 weeks	DLPFC/10 Hz	Figure-of-eight 70 mm air-cooled coil
Lee et al. (2016)	RCT	Patients with probable AD	5 sessions, weekly/6 weeks	DLPFC, L IFG, L STG, pSAC/10 Hz	Figure-of-eight 70 mm air-cooled coil
Rabey et al. (2013)	RCT	Patients with aMCI or mild to moderate probable AD	5 sessions, weekly/6 weeks	DLPFC, L IFG, L STG, pSAC/10 Hz	Figure-of-eight 70 mm air-cooled coil
Vecchio et al. (2022)	RCT	Patients with mild-to-moderate AD	5 sessions, weekly/6 weeks	DLPFC, L IFG, L STG, pSAC/10 Hz	Figure-of-eight magnetic coil
Qijuan et al. (2022)	RCT	Patients with AD	5 sessions, weekly/12 weeks	DLPFC/10 Hz	Figure-of-eight 70 mm air-cooled coil
Zhang et al. (2019)	RCT	Patients with mild to moderate AD	5 sessions, weekly/4 weeks	lDLPFC, LTL/10 Hz	Focal coil (MCF-B65 Butterfly coil; inner diameter, 35 mm; outer diameter, 75 mm; winding height, 12 mm)
Zhang et al. (2022)	RCT	Patients with AD	5 sessions, weekly/12 weeks	lDLPFC, LTL/1 Hz	-

Authors	Cognitive training intervention	Control	Measurement outcomes and time	Adverse effects	Main effect
Brem et al. (2013)	–	Usual care	ADAS-Cog/6 weeks	No	–
Brem et al. (2020)	Syntax, grammar, lexical meaning, categorization, action naming, episodic memory, spatial memory, spatial attention training	Cognitive training	ADAS-Cog/6 weeks	No	The combinatory intervention of repetitive transcranial magnetic stimulation and cognitive training showed significant cognitive improvement in patients with Alzheimer’s disease compared to the control group
Bagattini et al. (2020)	Episodic memory training	Cognitive training	MMSE/4 weeks, 12 weeks	No	An add-on generalization effect of real rTMS-CT in spatial reasoning
Jiang et al. (2022)	Mathematical calculations, agility drills, logic thinking, language, spatial memory, spatial attention training	Cognitive training	MMSE/4 weeks	No	rTMS-CT can effectively improve the cognitive function and psychobehavioral symptoms of patients
Lee et al. (2016)	Syntax, grammar, lexical meaning, categorization, action naming, episodic memory, spatial memory, spatial attention training	Cognitive training	ADAS-Cog, MMSE/6 weeks, 12 weeks	Mild headache and fatigability	The effect of rTMS-CT treatment remained steady or was even enhanced after the end of rTMS-CT treatment
Rabey et al. (2013)	Syntax, grammar, lexical meaning, categorization, action naming, episodic memory, spatial memory, spatial attention training	Cognitive training	ADAS-Cog/6 weeks	No	The improvement in the trained associative memory induced with rTMS was superior to that obtained with cognitive training alone
Vecchio et al. (2022)	Syntax, grammar, lexical meaning, categorization, action naming, episodic memory, spatial memory, spatial attention training	Cognitive training	ADAS-Cog/6 weeks, 40 weeks	No	rTMS-CT can improve cognitive functions in mild-to-moderate AD patients, with lasting effects up to 40 weeks after the end of treatment
Qijuan et al. (2022)	Multisensory stimulation training	Cognitive training	MMSE/12 weeks	No	rTMS-CT can effectively prevent the decline of patients’ cognitive function
Zhang et al. (2019)	Mathematical calculations, agility drills, logic thinking, language, spatial memory, spatial attention training	Cognitive training	ADAS-Cog, MMSE/4 weeks, 8 weeks	No	rTMS-CT significantly improved cognitive function and cortical metabolic ratios in patients with Alzheimer’s disease
Zhang et al. (2022)	Spatial memory, logic thinking training	Cognitive training	ADAS-Cog, MMSE/12 weeks	No	rTMS-CT can effectively improve the cognitive function and mental behavioral symptoms of patients, and the improvement effect of high frequency stimulation on cognitive function is more significant

DLPFC, dorsolateral prefrontal cortex; L IFG, left inferior frontal gyrus; L STG, left superior temporal gyrus; IPL, inferior parietal lobule; pSAC, parietal somatosensory association cortex; LTL, the left lateral temporal lob.

### 3.3 Risk of bias

**Figure 2:**
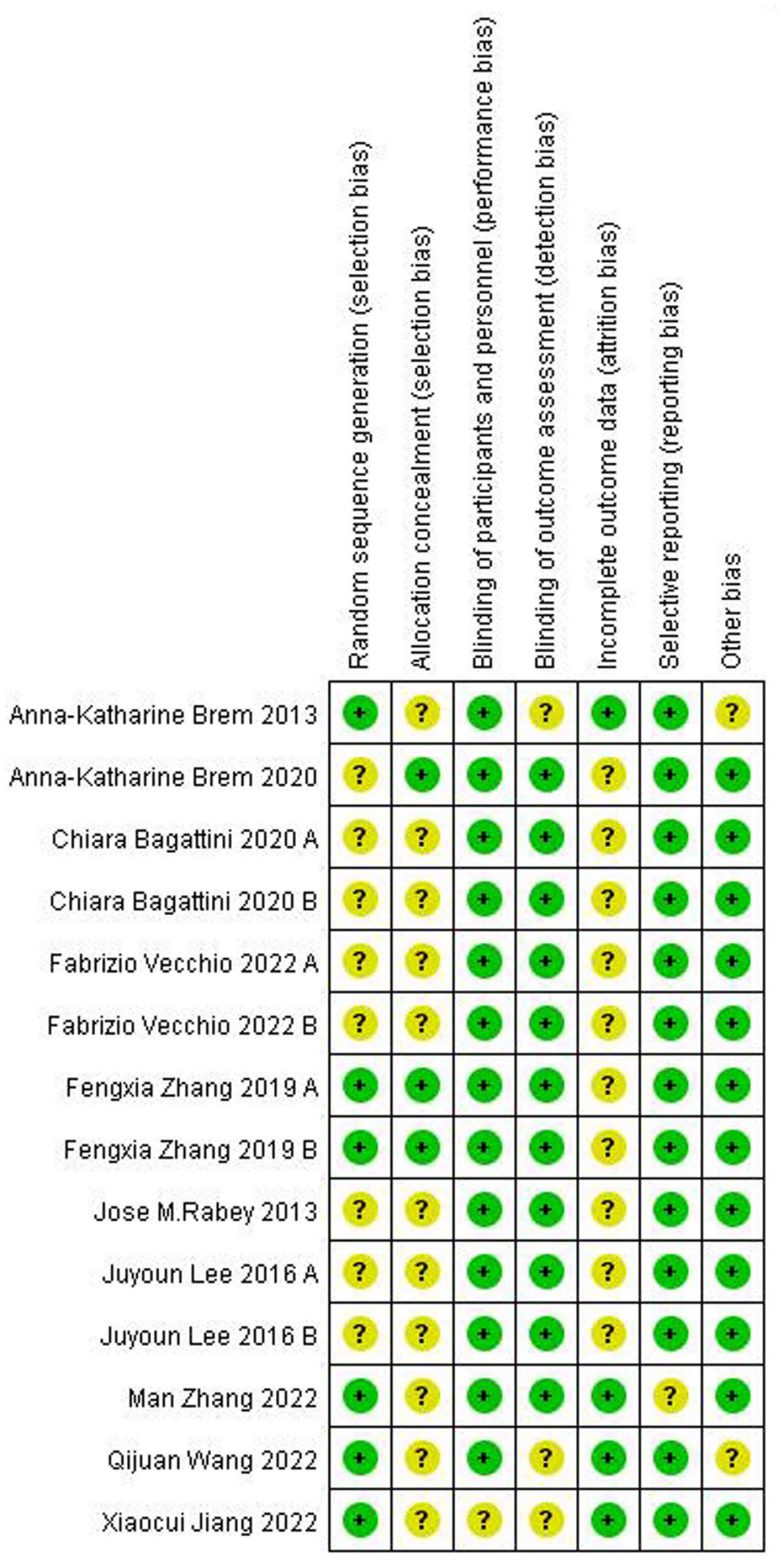
Risk of bias graph.

Figure 2 summarizes the risk of bias for all included studies. Overall, most included studies have an uncertain risk of bias in seven domains. Thirteen of fourteen trials mentioned the randomized allocation of participants. In all but three of the studies, the outcome assessors were blind to the group allocation, all trials applied to blind, and all trials reported reasons for withdrawal or dropout. The method of sequence generation was described in only six trials and allocation concealment was reported in only three trials.

### 3.4 The outcome of cognitive function

The results of rTMS-CT immediately after the intervention can be grouped according to assessment tools because different assessment tools can assess different cognitive functions. Therefore, the effects of rTMS-CT can be analysed according to different assessment tools. A study evaluated the effects of rTMS-CT with assessment tools such as MMSE, ADAS-Cog, and MoCA. They divided participants into three groups and found that rTMS-CT improved patients’ cognitive ability in the MMSE group. In the ADAS-Cog and MoCA groups, rTMS-CT training also significantly slowed down the rate of cognitive decline, respectively (Saitoh et al., 2022).

**Figure 3:**
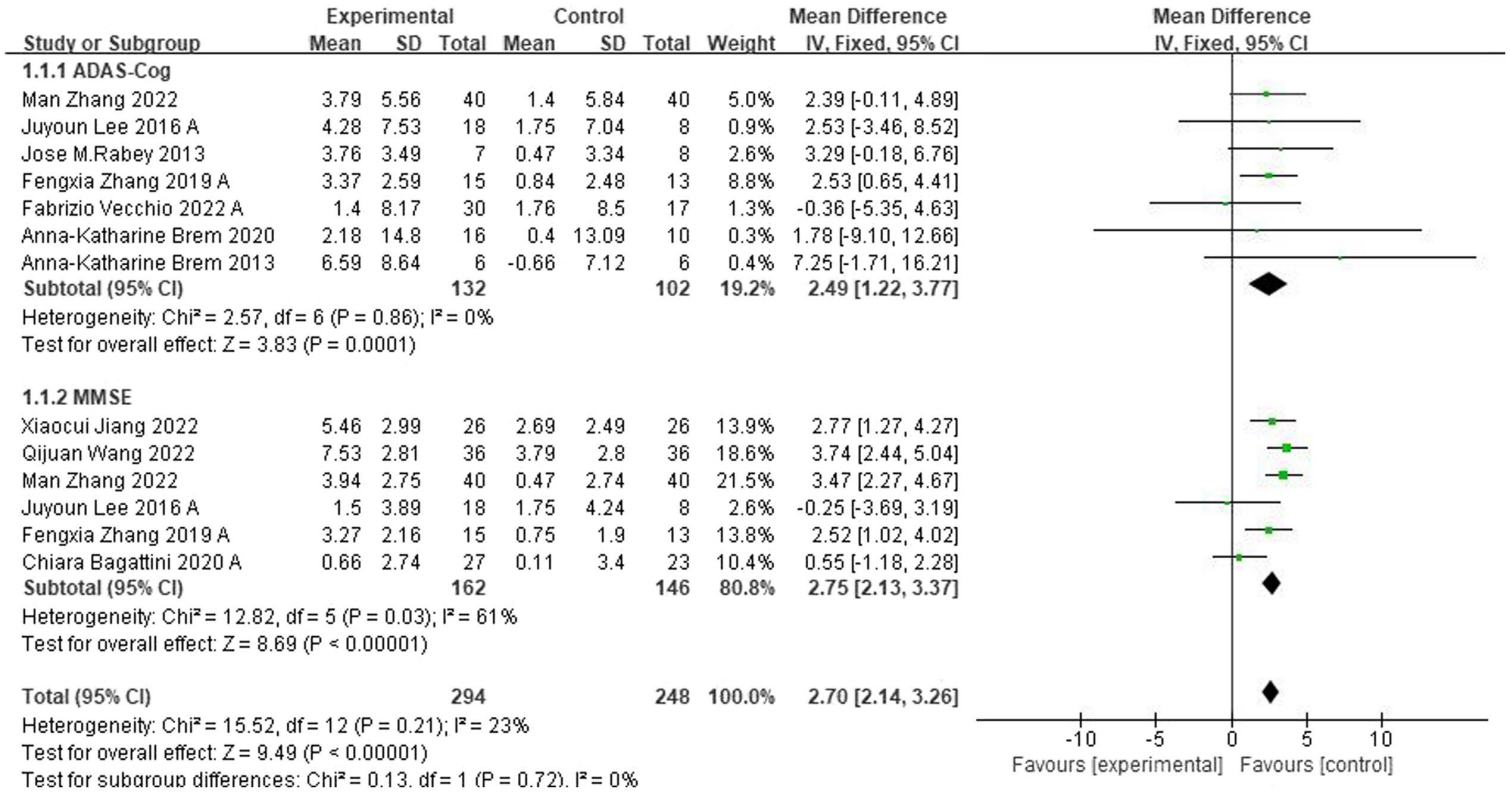
Forest plot of rTMS-CT vs. the control group by ADAS-Cog and MMSE immediately after the intervention.

Ten studies evaluated the immediate impact of rTMS-CT on cognitive function in patients with AD. Cognitive function was assessed using ADAS-Cog in seven studies (Brem et al., 2013, 2020; Rabey et al., 2013; Lee et al., 2016; Zhang et al., 2019, 2022; Vecchio et al., 2022) and MMSE in six studies (Lee et al., 2016; Zhang et al., 2019, 2022; Bagattini et al., 2020; Jiang et al., 2022; Qijuan et al., 2022). The results of both ADAS-Cog and MMSE showed that the cognitive function of the intervention group was superior to that of the control group, the mean effect size was 2.49 (95%CI, 1.22–3.77, *p* = 0.001, I^2^ = 0%) and 2.50 (95%CI, 1.45–3.54, *p* < 0.001, I^2^ = 61%), see Figure 3. Due to the high heterogeneity of MMSE, we used the random-effects model.

**Figure 4:**
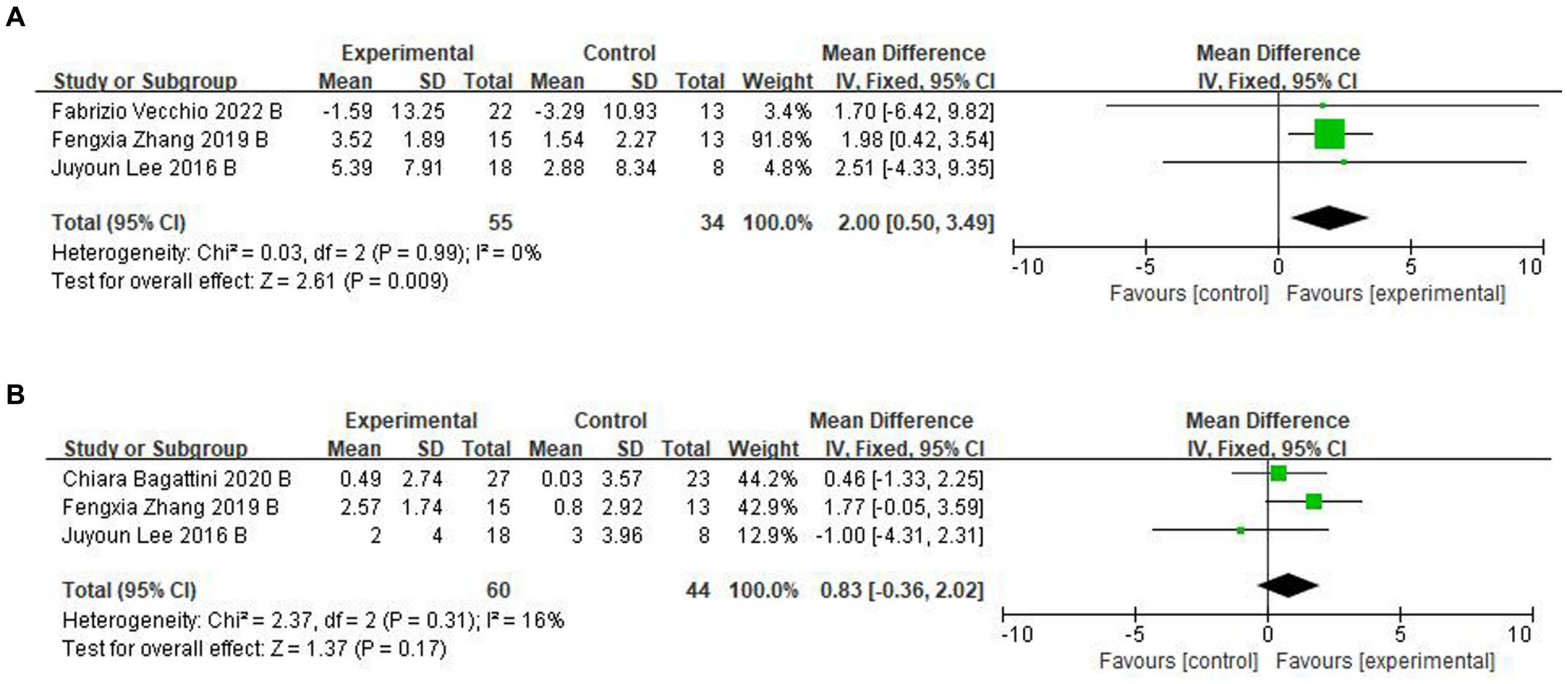
Forest plot of rTMS-CT vs. the control group by ADAS-Cog and MMSE at follow-up period. **(A)** ADAS-Cog. **(B)** MMSE.

For follow-up, four trials (Lee et al., 2016; Zhang et al., 2019; Bagattini et al., 2020; Vecchio et al., 2022) assessed the effects of rTMS-CT on cognitive function. In ADAS-Cog, there was a significant effect of 2.00 of cognitive function (95CI%, 0.50–3.49, *p* = 0.009, I^2^ = 0%), see Figure 4A, but in MMSE, this significant effect disappeared at 0.83 (95CI%, −0.36 to 2.02, *p* = 0.170, I^2^ = 16%), see Figure 4B.

### 3.5 Subgroup analysis by ADAS-Cog and MMSE

Several factors, including the site of stimulation, the frequency of stimulation, and the patient’s cognitive function, may influence the efficacy of rTMS-CT. Therefore, in studies, subgroup analyses of patients based on these factors are often required to evaluate the effects of rTMS-CT training more accurately. In addition, subgroup analysis of different sites and frequencies can help later studies determine which sites and frequencies should be selected for stimulation.

Three subgroups were constructed to identify the variables affecting the heterogeneity and cognitive function of ADAS-Cog or MMSE.

**Figure 5:**
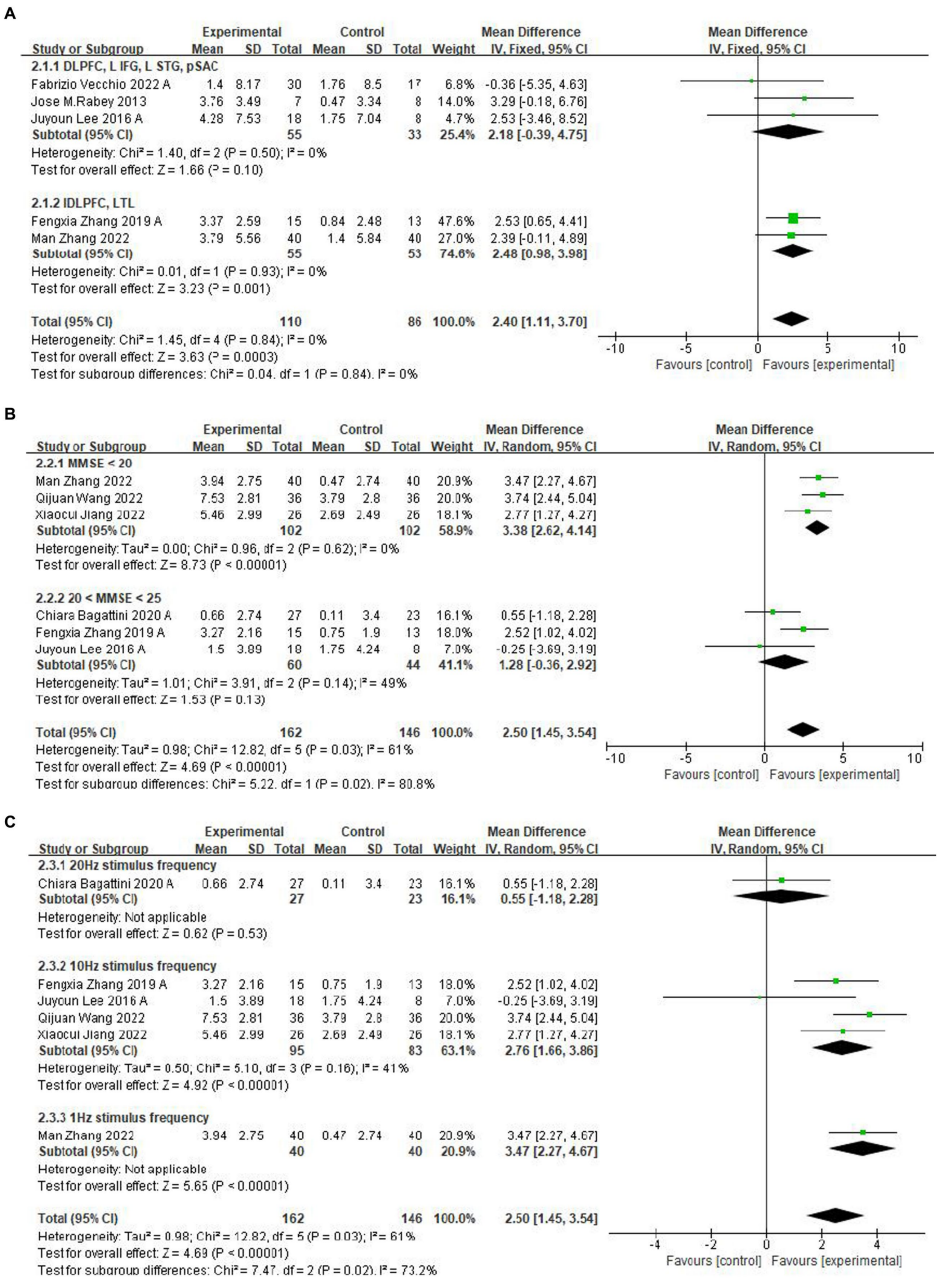
Subgroup analyses of rTMS-CT vs. the control group by ADAS-Cog and MMSE. **(A)** Stimulus area. **(B)** MMSE score. **(C)** Stimulus frequency.

First, a subgroup analysis based on the rTMS stimulation position was carried out, and the identical cognitive training treatments were applied across all chosen studies for each stimulation position. Compared with the left DLPFC and Left Temporal Lobe (LTL) (Zhang et al., 2019, 2022), the position of the DLPFC, Left Transverse Frontal Gyrus (LIFG), left superior temporal gyrus (LSTG) and Subcallosal Anterior Cingulate (pSAC) (Rabey et al., 2013; Lee et al., 2016; Vecchio et al., 2022) had less significant mean effects, see Figure 5A. Five studies were not included in this subgroup because they either selected to stimulate only the DLPFC position or selected less to stimulate the pSAC position (Brem et al., 2013, 2020; Bagattini et al., 2020; Jiang et al., 2022; Qijuan et al., 2022).

A subgroup analysis for different baseline means MMSE scores showed that lower baseline MMSE scores (Jiang et al., 2022; Qijuan et al., 2022; Zhang et al., 2022) have a more significant mean effect than higher baseline MMSE scores (Lee et al., 2016; Zhang et al., 2019; Bagattini et al., 2020), which were 3.38 (95%CI, 2.62–4.14, *p* < 0.001, I^2^ = 0%, MMSE <20) and 1.28 (95%CI, −0.36 to 2.92, *p* = 0.13, I^2^ = 49%, 20 < MMSE <25) (Figure 5B). The subgroup analysis of frequency reported a significant effect of the frequency of 1 Hz (Zhang et al., 2022) and 10 Hz (Lee et al., 2016; Zhang et al., 2019; Jiang et al., 2022; Qijuan et al., 2022), see Figure 5C.

**Figure 6:**
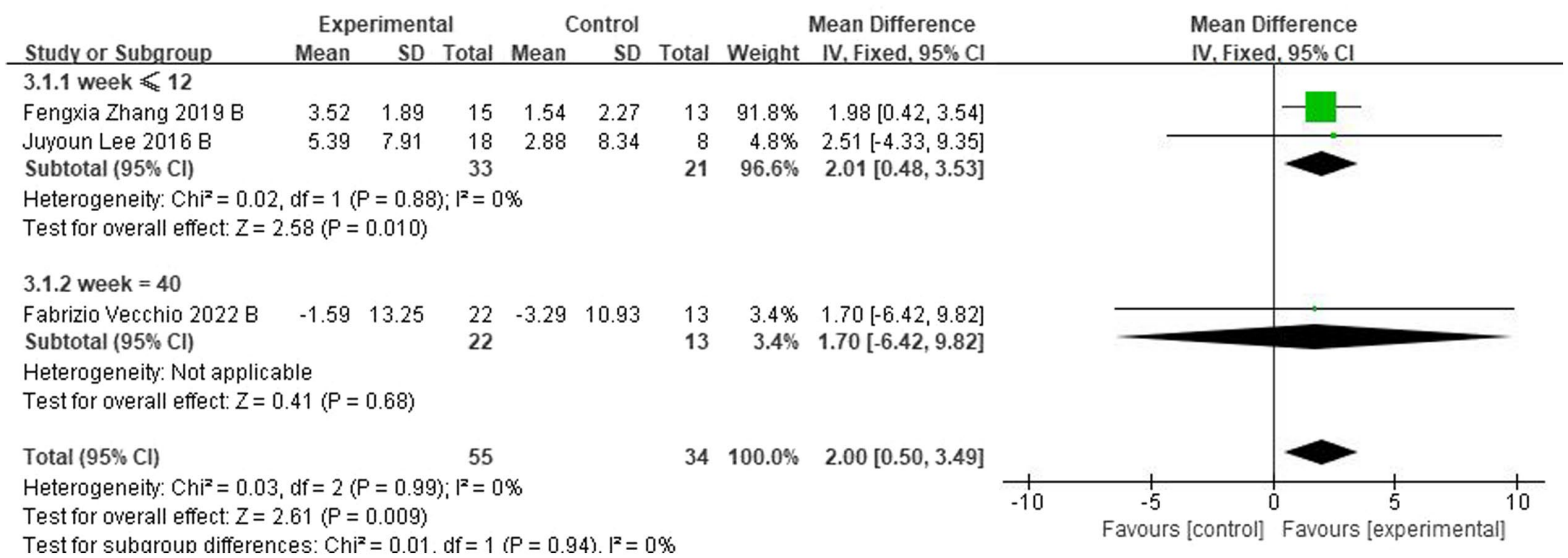
Subgroup analyses of rTMS-CT vs. the control group by ADAS-Cog: week <12 vs. week = 40.

For follow-up, the duration ranged from 8 to 40 weeks (Lee et al., 2016; Zhang et al., 2019; Vecchio et al., 2022). We classified the effects into short-term effects (≤12 weeks) and long-term effects (40 weeks). Subgroup analysis showed a mean effect size of 1.70 (95% CI, −6.42-9.82) for the longer duration effect for ADAS-Cog (Figure 6). The effect size for the short-term effect was 2.01 (95% CI, 0.48–3.53, I2 = 0%) (Figure 6). Significant transcranial magnetic stimulation effects were found in the shorter follow-up period.

### 3.6 Sensitivity analysis and publication bias

Sensitivity analysis was performed by deleting each test in turn. It was found that deleting any of the studies did not affect the conclusions, reflecting that the results were relatively stable.

Due to the small number of studies included (<10), a funnel plot was not used for publication analysis.

## 4 Discussion

There are many meta-analyses on treating Alzheimer’s disease with rTMS, but few comprehensive systematic reviews or meta-analyses on the combination of rTMS and CT. This systematic review and meta-analysis involving ten RCTs compared the effects of combined rTMS with CT versus CT on cognitive function in AD patients, indicating that treatment with rTMS-CT might be superior to CT.

Compared with the CT group, the intervention group significantly improved ADAS-Cog and MMSE scores in AD patients immediately after the intervention and at follow-up several weeks later. Subgroup analysis showed that treatment of AD patients with low MMSE scores resulted in more significant improvements in cognitive function. In addition, cognitive function continued to improve after the combination therapy ended, lasting for about 12 weeks. Moreover, rTMS are safe, with no serious adverse events occurring in the included studies and all minor adverse events resolved immediately after rTMS completion.

Previous meta-analyses have reported different results of the effect of rTMS-CT (Sitzer et al., 2006; Chu et al., 2021). In our study, rTMS-CT showed more significant cognitive improvement than rTMS. These results suggest that the combination of transcranial magnetic stimulation and CT may have additional effects and be more productive. Current studies have shown that rTMS-CT training can improve patients’ cognitive function in four aspects, namely, promoting neuroplasticity, improving brain network connectivity, promoting neuronal metabolism and blood flow, and affecting patients’ neurotransmitter levels (Wang et al., 2014; Chou et al., 2015). In general, consistent with previous studies, high-frequency rTMS had better therapeutic outcomes in treating cognitive function because high-frequency rTMS altered synaptic plasticity, increased the connection between the cerebral cortex, improved brain-derived neurotrophic factor (BDNF) levels (Coleman and Yao, 2003), stimulate the cerebral cortex, promote the local metabolism level of the brain, increase brain blood flow, reduce apoptosis and improve cognitive function (Tsai et al., 2020). High-frequency stimulation is usually excitatory to the stimulated cortical target, while low-frequency stimulation is usually inhibitory. It is important to select an optimal frequency. Both high-and low frequency are hypothesized to be associated with depression (Nicoletti et al., 2023). The effectiveness of rTMS in treatment and rehabilitation may be related to the depressive emotion of the AD olders. The previous literature also showed a large heterogeneity in the treatment effects of TMS programs in depressed and non-depressed older adults (Cappon et al., 2022). The majority of depressed patients experience significant benefits from the use of rTMS (Rachid, 2018). Previous studies have also shown positive results; however, future randomized controlled studies are needed to confirm the long-term safety and efficacy of maintaining rTMS in the treatment of depression.

Our meta-analysis showed that stimulation of the left DLPFC and LTL combined with corresponding cognitive training was superior to stimulation of the DLPFC and six brain regions like Broca’s area, Wernicke’s area, bilateral DLPFC and bilateral pSAC, which may be different with previous studies (Liao et al., 2015; Lin et al., 2019). Different researchers have come to different conclusions about whether bilateral DLPFC stimulation is superior to unilateral DLPFC stimulation. The above situation may be due to the small sample size of participants in the including studies. Further studies may be needed to explore rTMS stimulation targeting DLPFC and LTL positions.

Previous studies have found that the effectiveness of rTMS-CT is related to the patient’s gender, education level and age (Bagattini et al., 2020; Goldsworthy et al., 2020). This is because patients of different genders and stages of illness and so on may benefit to different degrees from rTMS-CT. This again reflects the importance of individualized care. What’s more, there are few adverse effects occurred especially one study showed mild headache and fatigue. rTMS-CT can be used as an effective non-pharmacological intervention.

Although this review and meta-analysis showed that rTMS-CT significantly improved cognitive function in patients with AD, the evidence included in this review was limited due to the lack of research on the effects of rTMS-CT on quality of life and satisfaction. In addition, although all studies are randomized controlled trials, the quality and methods of these studies are different, and the number of studies is small, which may affect the statistical power. However, we selected the most appropriate RCTs based on rigorous inclusion and exclusion criteria and all of the included studies were judged to have a mild risk of bias, which may be able to reduce the limitations imposed by the insufficient number of articles. What’s more, this study did not provide data on medication treatment and did not explore possible interactions between rTMS and medications and plasticity. Moreover, the duration of these studies is different, some of which are only a few weeks, while others are as long as months, which may affect the stability of the results. More rigorous research standards are needed to reduce heterogeneity. More large-sample clinical trials can be conducted in the future. More comprehensive assessments of the general health status, autonomy and quality of life of these older adults should be considered. And it is desirable to have a long-term follow-up plan to assess the durability and stability of the treatment response.

## 5 Conclusion

This study evaluated the effects and duration of improvement of rTMS-CT in patients with AD, suggesting that rTMS-CT can improve cognitive function in patients with AD compared with previous rTMS or CT alone, and the combination has a positive effect.

If rTMS-CT proves to be an effective treatment for AD, it could significantly impact the daily lives of patients and their caregivers. AD can profoundly affect a person’s ability to perform daily activities such as personal care, meal preparation, and communication. Improving cognitive function through rTMS-CT could enable patients to maintain their independence and quality of life for longer, reducing the burden on caregivers.

Furthermore, if rTMS-CT is proven effective, exploring its use in other neurodegenerative diseases or cognitive disorders could be beneficial. For example, Parkinson’s disease and traumatic brain injury can also result in cognitive impairment, and rTMS-CT may offer a non-invasive and safe treatment option for these patients.

In summary, the potential benefits of rTMS-CT for treating AD are promising, but further research is needed to confirm its effectiveness and optimal application. If proven effective, rTMS-CT could significantly impact the daily lives of patients and their caregivers and may also have potential applications in other cognitive disorders.

## Data availability statement

The raw data supporting the conclusions of this article will be made available by the authors, without undue reservation.

## Author contributions

GL: Conceptualization, Data curation, Formal analysis, Investigation, Methodology, Software, Validation, Writing – original draft, Writing – review & editing. BX: Conceptualization, Formal analysis, Investigation, Supervision, Visualization, Writing – original draft, Writing – review & editing. YG: Methodology, Supervision, Formal analysis, Validation, Visualization, Software, Writing – review & editing. XL: Conceptualization, Funding acquisition, Investigation, Project administration, Resources, Supervision, Validation, Visualization, Writing – review & editing.
